# Placenta-specific 9, a putative secretory protein, induces G2/M arrest and inhibits the proliferation of human embryonic hepatic cells

**DOI:** 10.1042/BSR20180820

**Published:** 2018-11-07

**Authors:** Cong Ouyang, Yi-Zhi Pu, Xu-Hui Qin, Jinhua Shen, Qing-Hua Liu, Liqun Ma, Lu Xue

**Affiliations:** Institute for Medical Biology and Hubei Provincial Key Laboratory for Protection and Application of Special Plants in Wuling Area of China, College of Life Sciences, South-Central University for Nationalities, 182 Minyuan Road, Hongshan District, Wuhan, 430074, China

**Keywords:** G2/M arrest, L02, Plac9, proliferation

## Abstract

**Background:** Placenta-specific 9 (*Plac9*) is a putative secreted protein that was first discovered in the context of embryogenesis. The expression pattern of *Plac9* during embryogenesis, together with the results of recent reports, suggest that *Plac9* may play a role in the liver development. The present study was conducted to investigate the secretory characteristics of *Plac9* and its potential role in liver cell physiology. **Methods:** Immunofluorescence was employed to identify the subcellular distribution of *Plac9*. Cellular proliferative activity was analyzed by MTT assay and cell colony formation. The cell cycle distribution of *Plac9* was analyzed by flow cytometry, and a functional analysis was performed using L02 cells following their stable infection with a lentivirus over-expressing *Plac9*. **Results:** *Plac9* is a novel protein that is localized to the cytoplasm and may be secreted through the classic endoplasmic reticulum-Golgi route. The overexpression of *Plac9* inhibits cell growth and induces G2/M phase arrest. **Conclusion:** Our findings reveal a novel role for *Plac9* in regulating cell growth.

## Introduction

As a temporary, dynamic, and complex organ, the placenta plays an interactive role between maternal mammals and their babies until delivery. The characteristics of the placenta are multifaceted, as this organ performs a host of important physiological functions during gestation, such as nutrient facility, gas exchange, barrier protection, and others [1–4]. Diverse placentae amongst mammals have been long regarded as a selective passive filter [5] that anchors the embryo or fetus to the uterine wall, provides O_2_ and nutrients, and removes CO_2_ and waste products throughout the entire embryogenesis process [6]. In addition, the placenta expresses a series of important immunological features. As a protective container, the placenta can alter maternal immune milieu by suppressing the host immune system or mimicking the response of the innate immune system to external stress [7].

Considering the physiological characteristics of the placenta, the placental proteins are presumably secreted into the maternal plasma, amniotic fluid, or other body fluid to perform their functions. For decades, many studies have indicated that the placenta is a rich source of secreted proteins. All such secretory factors are regulated by several tightly coordinated processes and play indispensable roles in a series of physiological processes [4]. One such example is placenta special gene 1 (*Plac1*), which is a protein that is selectively expressed in the embryo and plays a pivotal role in placental development by repressing the epithelial mesenchymal transition and decreasing cell migration and invasion [8,9]. Placenta special gene 8 (*Plac8*), which is also called onzin, was first identified in the placenta and was found to regulate a series of physiological process, including proliferation, division, differentiation, and apoptosis [10]. Placental protein 5 (*PP5*), a Kunitz-type serine proteinase inhibitor that is abundantly produced by the placenta, is thought to be an anti-coagulation factor that prevents the coagulation of extrinsic blood [11,12]. Placental protein 13 (*PP13*), which is a type of galectin produced only by the placenta, was revealed to be involved in placental implantation and maternal vascular remodeling [13,14]. In summary, these secreted proteins play important roles in a series of physiological processes.

One placental protein, placenta-specific 9 (*Plac9*) [15–22], was first identified by Galaviz-Hernandez et al. in a screen of mouse cDNA [15]. These authors found that *Plac9* is highly enriched in the placenta, and the sequence analysis of the promoter revealed that the expression of *Plac9* could be regulated by sets of relatively widely expressed transcription factors. Moreover, our previous study [23,24] on human embryogenesis also demonstrated that *Plac9* exhibited little embryonic expression in weeks 4 through 7 but was up-regulated by weeks 8–9. The expression pattern of *Plac9* in the human embryo implicated its role in embryonic development [24]. Other proteome-scaled studies revealed that *Plac9* was involved in liver protein interaction networks and may participate in liver development [17,18].

Although *Plac9* has been referred to in several studies, the detailed functions of *Plac9* have not been extensively studied until now. In the present report, we attempted to elucidate the function of *Plac9* in human liver cells. We discovered for the first time that *Plac9* is localized in cytoplasm, and might be secreted through the classic secretory pathway and inhibits the proliferation of normal human liver cells (L02) through the induction of G2/M phase arrest.

## Materials and methods

### Bioinformatic analysis

The secondary and tertiary structure of *Plac9* were predicted by PSIPRED [25] (http://bioinf.cs.ucl.ac.uk/psipred/) and SWISS-MODEL [26] (https://swissmodel.expasy.org/). The online programs Kyte-Doolittle Hydropathy Plot [27] (http://gcat.davidson.edu/DGPB/kd/kyte-doolittle.htm), SignalP 4.0 [28] (http://www.cbs.dtu.dk/services/SignalP/), and PSORTII [29] (https://psort.hgc.jp/) were employed to predict the secretory characteristics of *Plac9*.

### Plasmid construction and immunofluorescence staining

The entire protein-coding sequences of *Plac9*, thioredoxin (TRX) and chorionic somatomammotropin hormone 1 (CSH-1) were amplified from cellular cDNA by PCR using the primer pairs listed in Table 1. The PCR reaction was performed under the following conditions: 94°C for 10 min, 30 cycles of 94°C for 30 s, 58°C for 30 s, 72°C for 30 s, and then 72°C for 5 min. The purified PCR products were subcloned into the mammalian expression vector pCMV-3tag-8 (Invitrogen, Carlsbad, CA, U.S.A.) between the Xho I and Bam H1 sites. The sequences of all primers and the corresponding product sizes are shown in Table 1.

**Table 1:** Sequences of primers used in the present study

Primer name	Sequences	Product sizes (bp)
Plac9-3tag-S1	ATGCGGATCCCACCATGCGGCCCCTG	261 bp
Plac9-3tag-A1	GTACCTCGAGGAAGCCATCTCCGAGAAGG	
CSH1-3tag-S1	ATGCGGATCCATGGCTACAGGCTCCCGG	651 bp
CSH1-3tag-A1	GCATCTCGAGGAAGCCACAGCTGCCCTCCA	
hTRX-3tag-S1	ATCGGGATCCATGGTGAAGCAGATCGAGA	315 bp
hTRX-3tag-A1	CGCGCTCGAGGACTAATTCATTAATGGTGGC	
Plac9-F	GGCTGTGCAACGCCGTCTA	84 bp
Plac9-R	AGCAGGCCTTTCACCTCTGTC	
GAPDH-F	TGACTTCAACAGCGACACCCA	121 bp
GAPDH-R	CACCCTGTTGCTGTAGCCAAA	

16HBE cells grown on glass coverslips were transfected with the above mentioned plasmids. At 48-h post-transfection, 1 µg/ml brefeldin A (BFA, Sigma, St Louis, MO, U.S.A.) was added to 50% of the cells for 5 h. Then, the cells were washed twice with phosphate-buffered saline (PBS), fixed in 4% paraformaldehyde at room temperature for 20 min, and permeabilization in 0.5% Triton X-100 for 15 min at room temperature. The fixed cells were then blocked with 10% sheep serum at 4°C overnight and incubated with a monoclonal antibody against flag (1:1000) for 1 h at room temperature, followed by incubation with an anti-mouse IgG antibody (1:50). Next, the slides were embedded in an anti-fade aqueous mounting medium containing 1 µg/ml DAPI (Sigma, St Louis, MO, U.S.A.) and then evaluated and photographed under a laser-scanning LSM 510 confocal microscope (Carl Zeiss, Jena, Germany).

### Cell Culture, transient transfection, and clones that stably overexpress *Plac9*

QSG-7701, L02, and 16HBE cells were cultured in Roswell Park Memorial Institute Medium (RPMI-1640, HyClone, Waltham, MA, U.S.A.) supplemented with 10% fetal bovine serum (ScienCell, San Diego, CA, U.S.A.) and 1% Penicillin-Streptomycin (HyClone) in an atmosphere containing 5% CO_2_ at 37°C. SMMC-7721, SK-HEP-1, HepG2, Lm3, PLC, and 293T cells were cultured in Dulbecco’s modified Eagle Medium (DMEM, HyClone, Waltham, MA, U.S.A.) supplemented with 10% fetal bovine serum in an atmosphere containing 5% CO_2_ at 37°C. Lipofectamine™ 3000 (Invitrogen, Carlsbad, CA, U.S.A.) was used for the transient transfection of the expression plasmids according to the manufacturer’s protocols. Clones that stably overexpressed *Plac9* and the corresponding control were obtained from Genechem Co., Ltd, Shanghai, China.

### Cell proliferation assay and colony formation assay

Cells were treated with MTT (Sigma, St Louis, MO, U.S.A.) to detect the effects of *Plac9* overexpression on cell proliferation. In brief, the cells were cultured by seeding 10^3^ cells/well into a 96-well tissue culture plate. Then, 20 µl of the MTT reagent was added to each well for 4 h at 37°C. Next, 200 µl DMSO was added to each well, and the optical density was measured at 490 nm after 0, 24, 48, 96 and 120 h. Finally, the daily fold-change in the number of cells compared with the first day (od490/fold) was calculated in the control and experimental groups, and a curve representing cell proliferation was generated with time (day) on the *x*-axis and od490/fold on the *y*-axis.

In the colony formation assay, approximately 800 cells were plated and cultured in 60-mm culture dishes. Two weeks later, the colonies were fixed with ethanol and stained with 0.5% crystal violet. The clone formation rate was calculated as follows: clone counts/seeded cell counts × 100%.

### Cell cycle distribution analysis

The cell cycle distribution analysis was performed as previously described, with slight modification [30]. In brief, approximately 10^6^ L02 cells were pelleted and fixed with 70% ethanol for 1 h at 4°C. After resuspending the cells in staining buffer (2 mg/ml PI: 10 mg/ml RNase: 1 × PBS = 25: 10: 1000), the cell cycle distribution was assessed using a BD FACS caliber flow cytometer (BD Biosciences, San Jose, CA, U.S.A.) according to the manufacturer’s instructions. A bar chart of cell cycle distribution was generated with cell phase (G1, S, and G2/M) on the *x*-axis and the percentage (%) on the *y*-axis. Each bar represents the mean of a triplicate experiment ± standard deviation (SD). *P*<0.05 represents statistical significance.

### Reverse transcription and quantitative real-time PCR

The mRNA expression of *Plac9* was detected by quantitative real-time PCR analysis using a QuantStudio® 5 Real-Time PCR System (Applied Biosystems, Foster City, CA, U.S.A.) according to the manufacturer’s instructions. In brief, the total RNA was extracted from the cells using an RNA extraction kit (Bioteke, Beijing, China), and cDNA was synthesized using a cDNA synthesis kit (Thermo Fisher Scientific, San Jose, CA, U.S.A.) according to the manufacturer’s instructions. Then 5 ng of cDNA was amplified with the indicated primers (Table 1) using the SYBR Green Real-time PCR Master Mix (Toyobo, Osaka, Japan). The thermal cycling conditions were as follows: 95°C for 30 sec, 45 cycles of 95°C for 5 sec, and 60°C for 30 sec, followed by a melting curve analysis using the default program of the QuantStudio® 5 Real-Time PCR machine. The mRNA expression levels of *Plac9* in the hepatic cell lines were calculated relative to that of GAPDH (internal control) using the 2^−ΔΔ*C*t^ method.

### Western blot analysis

Cells were collected and lysed in protein lysis buffer (Beyotime, Shanghai, China) supplemented with PMSF (Beyotime, Shanghai, China) and a protease inhibitor cocktail (Bimake, Houston, TX, U.S.A.). In brief, protein samples (20 μg) were separated via 12% SDS–PAGE and transferred to PVDF membranes. The membranes were blocked with 5% non-fat milk. Next, the membranes were incubated with the indicated antibodies (Cell Signaling Technology, Beverly, MA, U.S.A.) following the manufacturer’s instructions. Immunoreactive bands were visualized using the ECL reagents (Thermo Fisher Scientific, San Jose, CA, U.S.A.) and quantitatively assessed using the ImageLab® software (Bio-Rad, Hercules, CA, U.S.A.). Protein expression was normalized to that of β-actin, and the results represent the mean of three independent experiments.

### Data analysis

Differences between groups were analyzed using the Student’s t-test. All experiments were repeated at least three times. The results are expressed as the mean ± SD. *P*<0.05 indicates a significant difference.

## Results

### Characterization of the *Plac9* gene

**Figure 1:**
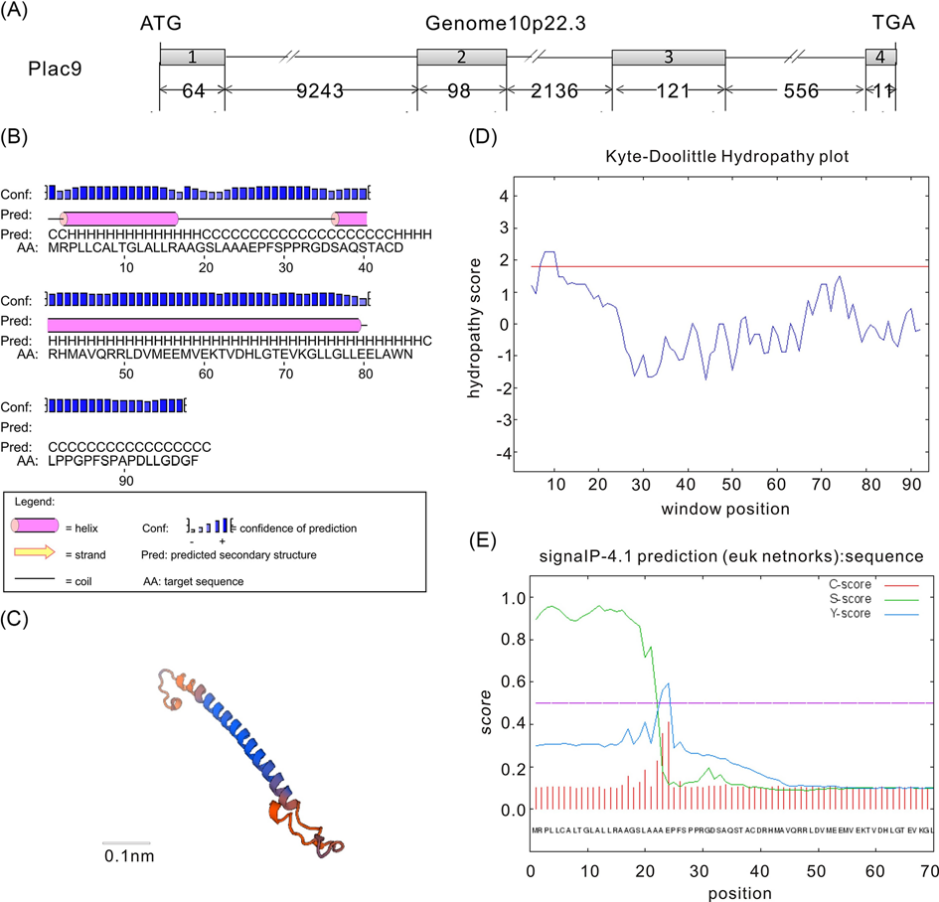
Genomic structure, protein structure, and bioinformatic analysis of *Plac9* (**A**) The genomic information of *Plac9*. Gray boxes and dark lines denote exons and introns, respectively. The start codon (ATG) and stop codon (TGA) of protein translation are labeled. The numbers underneath represent nucleotide length. (**B**) The secondary structure prediction of *Plac9*. The gray columns and dark lines depict helices and coils, respectively. (**C**) The tertiary structure prediction of *Plac9*. Scale bar is 0.1 nm. (**D**) The signal peptide prediction of *Plac9*. The *x*-axis and *y*-axis represent the number of amino acid residues and the possibility of cleavage, respectively. (**E**) The hydropathy plot of *Plac9*. The *x*-axis and *y*-axis indicate the number of amino acid residues and hydrophobicity, respectively.

The human *Plac9* gene was first identified in our previous study on embryogenesis and exhibited up-regulated expression in embryos from weeks 4 to 9 [24]. A survey of the human genomic database on the NCBI website indicated that the *Plac9* gene is located on human chromosome 10q22.3, which contains 4 exons and 3 introns and encodes a protein with 97 amino acid (Figure 1A). The secondary and tertiary structure analysis indicated that human *Plac9* protein contains two helices and two coils (Figure 1B,C). The online software SignalP 4.0 indicated that there is a signal peptide in the N-terminus of the *Plac9* protein from aa 1 to 23 (Figure 1D). In addition, Kyte-Doolittle hydropathy analysis revealed that the middle sequence of *Plac9* is hydrophilic, whereas the N-terminus contains a potential transmembrane region, which may indicate the potential transportation of *Plac9* to the extracellular region (Figure 1E). Moreover, the PSORT II program predicted the following probabilities of the subcellular localization of *Plac9*: extracellular, 66.7%; Golgi, 11.1%; vacuolar, 11.1%; and endoplasmic reticulum (ER), 11.1%. Taken together, these bioinformatic analyses suggest that *Plac9* may be a secreted protein.

### Subcellular localization of the *Plac9* protein

**Figure 2:**
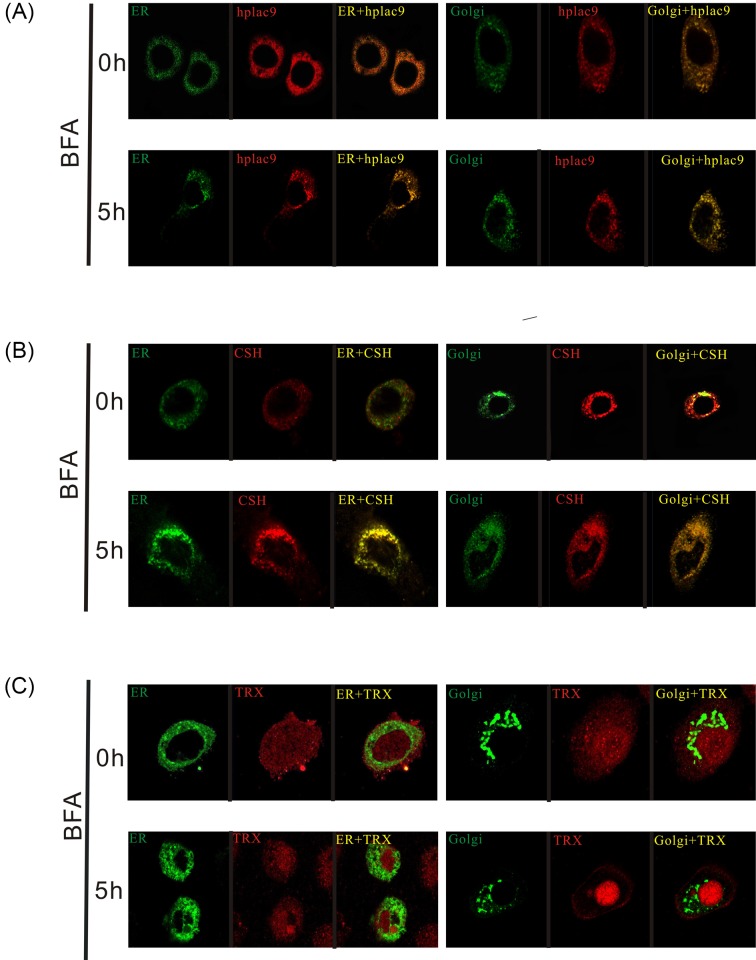
Secretory pathway analysis of the *Plac9* protein (**A**) Co-localization analysis of *Plac9* with the ER in the left panel or the Golgi apparatus in the right panel with or without BFA treatment. (**B**) Co-localization analysis of CSH-1, a classical secreted protein with the ER or Golgi apparatus with or without BFA treatment. (**C**) TRX served as a non-classical secreted protein control.

To explore the characteristics of *Plac9*, we first examined the subcellular distribution of *Plac9*. As shown in Figure 2A in the images from confocal microscopy, *Plac9* was significantly localized to the cytoplasm. To further investigate the localization of *Plac9*, the EYFP-ER, EYFP-Golgi, and *Plac9*-FLAG plasmids were employed to visualize the ER apparatus, Golgi apparatus, and *Plac9* protein, respectively. Then, these markers were compared with two additional markers, TRX, which has been documented as a non-classical protein that cannot co-localize with the ER and Golgi [31], and CSH-1, a classical secretory placental hormone that does co-localize with the ER and Golgi [32]. In addition, BFA, an inhibitor of ER-to-Golgi protein transport, was applied to verify the role of the Golgi in the subcellular localization of *Plac9*.

As shown in Figure 2A, the *Plac9* protein significantly co-localized with the ER. However, the co-localization was not complete. *Plac9* was also expressed outside of the ER in the cytoplasm, which indicates that *Plac9* can be transported out of the ER lumen. The expression distribution of *Plac9* was similar to that of CSH-1 (Figure 2B). After 5 h of treatment with BFA, it was observed that all *Plac9*, as well as CSH-1, co-localized completely and were expressed only in the ER, which indicates that the transport from the ER to the Golgi had been effectively blocked by BFA (Figure 2A,B). In contrast, TRX, the well-documented non-classical secreted protein that was distributed all around the cell, could not co-localize with these two organelles, with or without BFA treatment (Figure 2C). In conclusion, the distribution of *Plac9* is similar to that of the classical secretory protein CSH-1, which strongly suggests that *Plac9* co-localizes with the ER and Golgi apparatus in the manner of classical secretory proteins.

### The expression pattern of *Plac9* in hepatic cell lines

**Figure 3:**
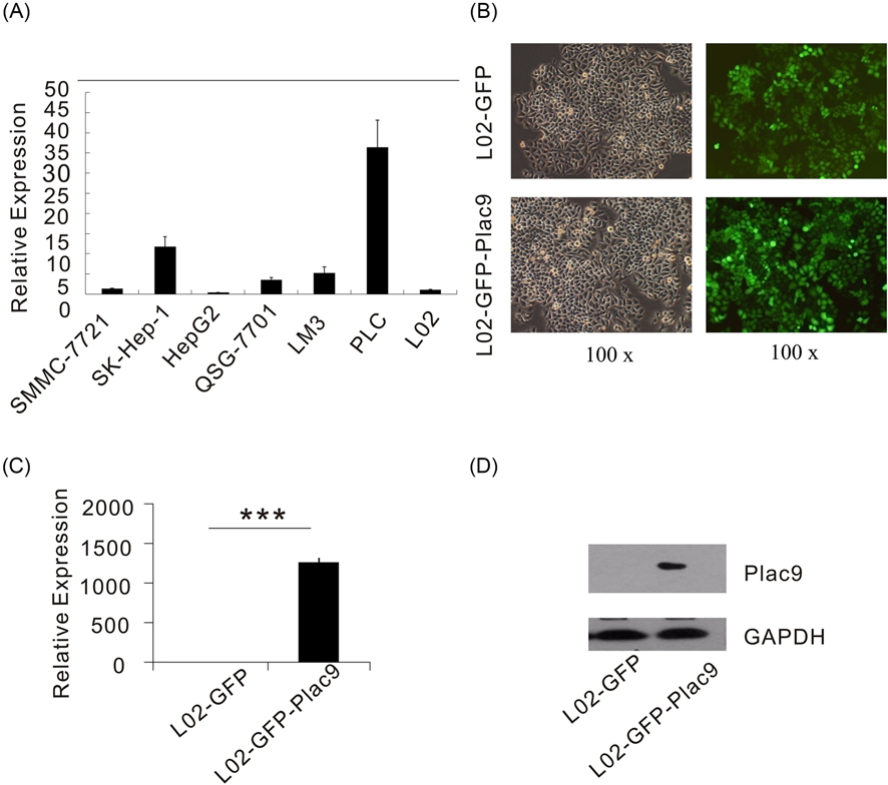
The expression pattern of *Plac9* in hepatic cell lines and the generation of stable *Plac9*-expressing L02 cells via lentiviral transduction (**A**) The relative mRNA expression level of *Plac9* in SMMC-7721, SK-HEP-1, HEP-G2, QSG-7701, Lm3, PLC, L02 cells. (**B**) Detection of GFP via fluorescence microscopy after the lentiviral infection (green, *Plac9*; ×100 view) Left panel: bright sight. Right panel: dark sight. (**C**) The relative mRNA expression levels of *Plac9* in L02-GFP and L02-GFP-*Plac9* cells after the lentiviral infection. (**D**) Protein expression levels of *Plac9* in L02-GFP and L02-GFP-*Plac9* cells after the lentiviral infection. GAPDH served as an internal control. ^***^P<0.001.

Recent proteome-scale studies reported that *Plac9* is involved in liver protein interaction networks and may participate in liver development [17,18]. To further investigate the biological function of *Plac9*, the expression levels of *Plac9* were detected in a series of hepatic cell lines, including SMMC-7721, SK HepG2, and L02. As shown in Figure 3A, the expression level of *Plac9* was higher in the primary liver carcinoma cell line PLC relative to human hepatoma cells SK-HEP-1, SMMC-7721, Lm3 and the normal liver cell line QSG-7701; moreover, in HEP-G2 cells and the human embryonic liver cell line L02, *Plac9* expression was almost undetectable. This variation in expression patterns reflects that *Plac9* may play different roles in various hepatic cell lines. The L02 cell line, a human normal hepatocyte cell line, was chosen for further functional exploration. A stable *Plac9*-expressing cell line (L02-GFP-*Plac9*) was generated by the transfection of the lentiviral *Plac9* vector into L02 cells (Figure 3B). qPCR analysis and Western blot analysis confirmed that the L02-GFP-*Plac9* cells exhibited significantly increased mRNA and protein levels of *Plac9* when compared with the control vehicle (Figure 3C,D).

### Overexpression of *Plac9* inhibits cell proliferation and colony formation

**Figure 4:**
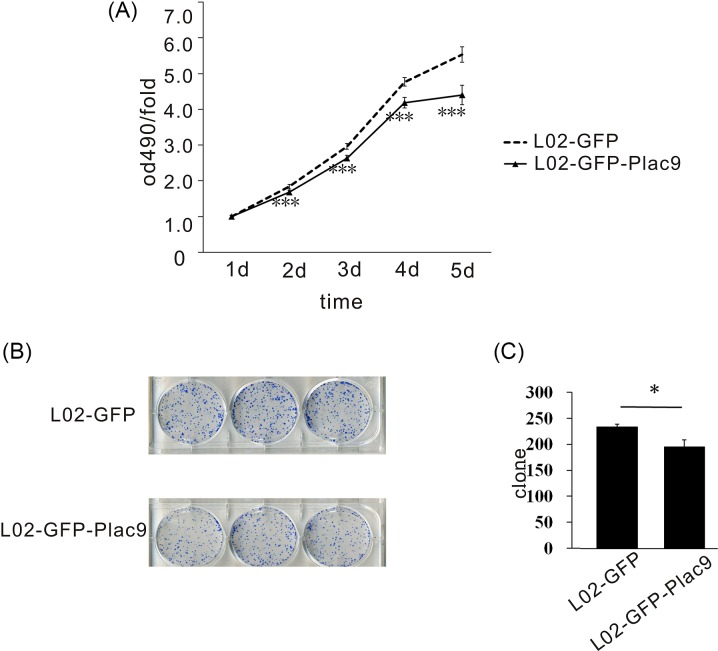
The overexpression of *Plac9* inhibited cellular proliferation and colony formation (**A**) The overexpression of *Plac9* inhibited cellular proliferation. (**B,C**) The overexpression of *Plac9* inhibited colony formation. ^*^P<0.05, ^***^P<0.001.

Using the L02-GFP-*Plac9* cell line, the function of *Plac9* in normal liver cells was further explored by MTT assay. Compared with L02-GFP, the L02-GFP-*Plac9* cells exhibited a decreased proliferative capacity (Figure 4A). Furthermore, *in vitro* colony formation assays demonstrated that the frequencies of colony formation of the L02-GFP-*Plac9* cells were statistically lower than those of the L02-GFP cells (Figure 4B,C). These results indicate that *Plac9* may inhibit cell proliferation.

### Plac9 overexpression redistributed the cell cycle

**Figure 5:**
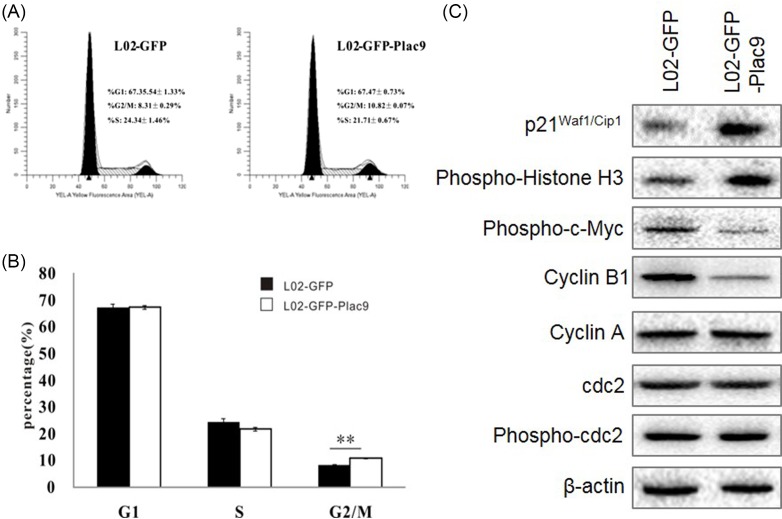
The overexpression of *Plac9* induced G2/M phase arrest (**A,B**) The cell cycles of L02-GFP and L02-GFP-*Plac9* cells were analyzed by FACS. (**C**) Western blot analysis of the protein levels of c-Myc, cyclin B1, cdc2, Myt1, and p21^Waf1/Cip1^, and Histone H3 in L02-GFP and L02-GFP-Plac9 cells. β-actin was used as a loading control. ^**^P<0.01.

To determine the phase at which Plac9 inhibits cell growth in the cell cycle progression of L02 cells, FACS analysis was employed to evaluate the impact of *Plac9* expression on cell cycle progression. *Plac9* overexpression resulted in a higher percentage of cells at the G2/M phase, which displayed G2/M phase arrest (Figure 5A,B). Subsequently, alterations of cell cycle-related markers were detected. Western blot analysis of G2/M phase-related proteins, including cyclin A, cyclin B1, cdc2, c-Myc, Histone H3, p21^Waf/Cip1^, and others (Figure 5C) revealed that the L02-GFP-*Plac9* cells expressed lower levels of Phospho-c-Myc (Ser62) and cyclin B1, and higher levels of p21^Waf/Cip1^ and phospho-Histone H3 (Ser10) compared with the control. These data confirmed that *Plac9* may affect the proliferation of L02 cells by altering the cell cycle and the expression of cell cycle-related proteins.

## Discussion

The human placenta is comprised of active secretory tissue [2,5]. A series of placenta-enriched secreted proteins play vital roles in innate immunity, material exchange, and embryo development [4]. *Plac9* is a placenta-enriched protein that was first identified from a collection of 15000 mouse genes [15]. Although several studies have indicated the potential role of *Plac9* [15–22], the exact function of *Plac9* is still unclear. In our previous study on human embryogenesis [24], it was found that the up-regulated expression pattern of *Plac9* was consistent with embryogenesis, particularly the organ development stage. Further proteome-scale studies on the human liver revealed that *Plac9* was also involved in protein interaction networks, which implicated its potential role in liver development [17].

First of all, we explored the subcellular characteristics of *Plac9*. The bioinformatic prediction indicated that *Plac9* may be extracellularly localized. In general, there are two distinct secretory pathways. Classical secretory proteins are translated in the ER and then transported to the Golgi apparatus for further modification, and then they are finally secreted to the extracellular space through the release of secretory vesicles [33]. Non-classical secreted proteins, which lack an N-terminal signal sequence, may bypass the ER or Golgi and other secretory granules and use an alternative secretory pathway [34]. In the present study, the confocal microscopy data demonstrated that *Plac9* was distributed in the cytoplasm and co-localized with the ER and Golgi, which was partly consistent with our previous bioinformatic prediction.

Next, we explored the function of *Plac9* in hepatic cell lines. The quantitative real-time PCR data revealed that *Plac9* was expressed to varying degrees in a series of liver cell lines. To assess the functions of *Plac9* in embryonic liver development, the fetal liver cell line L02 was employed to establish a *Plac9*-overexpressing stable cell line. Using this stable cell line, it was demonstrated that the overexpression of *Plac9* inhibited cellular proliferation and colony formation.

It is well known that the inhibition of cell growth is typically accompanied by cell cycle redistribution [35–38]. The FACS data revealed that the over-expression of *Plac9* in the L02 cell line resulted in an increase in the G_2_/M-phase population. During the cell cycle progression through the G_2_/M checkpoint, a series of cell cycle regulators are involved. Traversing the G2/M checkpoint to initiate mitosis requires two important regulators, cyclin B1 and cdc2 [39–41]. Phosphorylated c-Myc binds to the promoter of cyclin B1, resulting in increased cyclin B1 promoter activity, which is critical for the completion of the G2/M transition [42]. Subsequently, cyclin B1 is degraded during mitosis, particularly at the transition from metaphase to anaphase [43]. The phosphorylation of cdc2 at Tyr15 and Thr14 is known to be a critical regulatory step that inhibits cdc2 activity and causes G2/M arrest [44,45]. In addition to cyclin B1 and cdc2, the cyclin-dependent kinase (CDK) inhibitor p21^Waf1/Cip1^ negatively modulates G2/M cell-cycle progression by binding to the cyclin-CDK complex [35]. Cyclin A promotes mitotic entry [46]. The phosphorylation of Histone H3 at Ser 10 is reported to be correlated with chromosome condensation during mitosis [47,48].

Our Western blot analysis demonstrated that the overexpression of *Plac9* reduced c-Myc phosphorylation and subsequently decreased cyclin B1 levels, which inhibited the cdc2/cyclin B1 kinase activity and caused G2/M arrest. Meanwhile, the elevated protein levels of p21^Waf1/Cip1^ also accelerated the blocking of G2/M cell-cycle progression in L02-GFP-*Plac9* cells. Furthermore, the up-regulated phosphorylated Histone H3 and degraded cyclin B1 indicated that L02-GFP-*Plac9* cells may be arrested in the M phase rather than G2 phase. Nevertheless, we did not detect any change in cdc2 or cyclin A. Thus, taken together, our data suggest that the *Plac9* overexpression-induced inhibition of the proliferative capacity of hepatic cells is associated with G2/M arrest and the regulation of phosphorylated c-Myc, cyclin B1, phosphorylated histone H3 expression, and p21^Waf1/Cip1^.

Above all, our study revealed for the first time the basic biological characteristics of *Plac9*. It was found that *Plac9* is a cytoplasm-located protein and may be secreted via the classical ER-Golgi route. Moreover, to our knowledge, these findings have revealed for the first time that *Plac9* may play a pivotal role in the proliferation of liver cells by altering a series of cell cycle-related proteins. However, the understanding of *Plac9* remains poor. The secretory characteristics of *Plac9* and the molecular mechanism underlying its regulation of cellular proliferation warrant further investigation.

## Abbreviation

BFA: brefeldin A
cdc2: cell division cycle 2
CDK: cyclin-dependent kinase
CSH-1: chorionic somatomammotropin hormone 1
DAPI: 4′,6-diamidino-2-phenylindole
ER: endoplasmic reticulum
GAPDH: glyceraldehyde-3-phosphate dehydrogenase
GFP: green fluorescent protein
PBS: phosphate-buffered saline
Plac9: Placenta-specific 9
qPCR: quantitative PCR
TRX: thioredoxin
SD: standard deviation
